# Analyzing and Characterizing the Chloroplast Genome of* Salix wilsonii*

**DOI:** 10.1155/2019/5190425

**Published:** 2019-07-15

**Authors:** Yingnan Chen, Nan Hu, Huaitong Wu

**Affiliations:** Key Laboratory of Forest Genetics & Biotechnology of Educational Department of China and of Jiangsu Province, College of Forestry, Nanjing Forestry University, Nanjing 210037, China

## Abstract

*Salix wilsonii* is an important ornamental willow tree widely distributed in China. In this study, an integrated circular chloroplast genome was reconstructed for* S. wilsonii* based on the chloroplast reads screened from the whole-genome sequencing data generated with the PacBio RSII platform. The obtained pseudomolecule was 155,750 bp long and had a typical quadripartite structure, comprising a large single copy region (LSC, 84,638 bp) and a small single copy region (SSC, 16,282 bp) separated by two inverted repeat regions (IR, 27,415 bp). The* S. wilsonii* chloroplast genome encoded 115 unique genes, including four rRNA genes, 30 tRNA genes, 78 protein-coding genes, and three pseudogenes. Repetitive sequence analysis identified 32 tandem repeats, 22 forward repeats, two reverse repeats, and five palindromic repeats. Additionally, a total of 118 perfect microsatellites were detected, with mononucleotide repeats being the most common (89.83%). By comparing the* S. wilsonii* chloroplast genome with those of other rosid plant species, significant contractions or expansions were identified at the IR-LSC/SSC borders. Phylogenetic analysis of 17 willow species confirmed that* S. wilsonii* was most closely related to* S. chaenomeloides* and revealed the monophyly of the genus* Salix*. The complete* S. wilsonii* chloroplast genome provides an additional sequence-based resource for studying the evolution of organelle genomes in woody plants.

## 1. Introduction

In plant, chloroplast is an essential organelle with its own genome and servers as the metabolic center involved in photosynthesis and other cellular functions, including the synthesis of starch, fatty acids, pigments, and amino acids [1]. In most land plants, the chloroplast (cp) genome has a circular quadripartite structure, comprising four major segments: two inverted repeat regions (IRa and IRb), a large single copy (LSC) region, and a small single copy (SSC) region. The gene content and order are highly conserved among land plants, with most genes involved in photosynthesis, transcription, and translation [1, 2]. Despite the overall conservation, during evolution, cp genomes have undergone extensive rearrangements within and between plant species, including gene/intron gains and losses, expansion and contraction of the IRs, and inversions [2, 3]. This information, which is revealed by comparisons of cp genomes, has been especially valuable for plant phylogenetic and evolutionary studies. The elucidation of the variations among cp genomes has also contributed to the characterization of chloroplast-to-nucleus gene transfer, which plays an important role in the evolution of eukaryotes. Furthermore, the uniparental inheritance of the cp genome (usually maternal in angiosperms and paternal in gymnosperms), accompanied by the general lack of heteroplasmy and recombination, has enabled researchers to evaluate the relative influences of seed and pollen dispersal on total gene flow [4].

In addition to increasing the available information from functional and evolutionary perspectives, chloroplast genomics research has important implications for chloroplast transformation, which has advantages over nuclear transformation, including enhanced transgene expression and lack of transgene escape via pollen [5]. Because of the rapid and cost-effective development of high-throughput sequencing technology, more than 2,000 complete cp genomes of land plants are now available in the NCBI Organelle Genome Resources database (http://www.ncbi.nlm.nih.gov/genome/organelle/). Since the first report by Ferrarini et al. [6], the third-generation PacBio RS platform has been applied for sequencing the cp genomes of many plant species [7–11], confirming the utility of PacBio RS data for the sequencing and* de novo* assembly of cp genomes.

Willows (*Salix* L.) are economically and ecologically important woody plants because of their considerable biomass production and resistance to environmental stresses [12, 13]. Moreover,* Salix* L. represents one of the most taxonomically complex genera of flowering plants and comprises 330-500 species, including tall trees, shrubs, bushes, and prostrate plants [14, 15]. Despite the high species diversity, the cp genomes of only 15* Salix* species have been sequenced (i.e., nine shrub and six tree species).* Salix wilsonii*, which is commonly referred to as Ziliu in China, is a deciduous tree that can grow up to 13 m tall. It is a representative of section* Wilsonia*, which consists of 15 species [16]. Being native to China,* S. wilsonii* is widely distributed in Huanan, Hubei, Jiangxi, Anhui, Zhejiang, and Jiangsu provinces [16]. Additionally, one-year-old branchlets of this tree have a dull brown surface and its young leaves appear slightly red. These attractive characteristics make* S. wilsonii* an important ornamental plant in Middle and Eastern China. As part of an ongoing project to sequence the* S. wilsonii* nuclear genome, we assembled and characterized the cp genome by screening for chloroplast reads in the data generated with the PacBio RSII platform. Analyzing the* S. wilsonii* cp genome will help researchers resolve the phylogenetic relationships among* Salix* species and clarify the evolution of cp genomes in the family* Salicaceae*.

## 2. Materials and Methods

### 2.1. Chloroplast Reads Extraction and Assembly

Fresh and young leaves were collected from a single* S. wilsonii* tree on the campus of Nanjing Forestry University, Jiangsu, China. Total DNA was extracted using the CTAB method [17] and subjected to whole-genome sequencing with the PacBio RSII platform (NestOmics, Wuhan, China). The sequencing library was constructed according to the 20-kb template preparation protocol [18]. Approximately 31 Gb of clean data including 2.8 M high-quality long reads were obtained (unpublished data). By mapping the high-quality reads to the land plant cp genomes available in the NCBI Organelle Genome Resources database,* S. wilsonii* chloroplast reads were extracted with a BLASTN algorithm (e-value of 1e^−5^). The extracted reads were first filtered to remove repetitive and shorter reads (<15,000bp). The remaining reads were error-corrected, trimmed, and assembled* de novo *using Canu version 1.4 [19] with the corOutCoverage=100, genomeSize=150 Kb and all other parameters set as default. The complete* S. wilsonii* cp genome sequence was deposited in the GenBank database (accession number: MK603517).

### 2.2. Chloroplast Genome Annotation and Sequence Analyses

The resulting FASTA file containing the assembled* S. wilsonii* cp genome sequence was annotated with the DOGMA (https://dogma.ccbb.utexas.edu/). The percent identity cutoff for protein-coding genes and RNAs was set to 60 and 85, respectively. The start and stop codons were manually corrected to match the gene predictions. The identified tRNA genes were confirmed with tRNAscan-SE 1.21 [20]. Consequently, a circular cp genome map was obtained with the OGDRAW version 1.1 (http://ogdraw.mpimp-golm.mpg.de/), and the extent of the repeat and single copy regions was specified manually.

The GC content and relative synonymous codon usage (RSCU) values were determined with MEGA 7.0.21 [21]. Microsatellite or simple sequence repeats (SSRs) with core motifs of 1-6 bp were detected with the Perl script program MISA (http://pgrc.ipk-gatersleben.de/misa/). The minimum repeat number was set to 8, 6, 4, 3, 3, and 3 for mono-, di-, tri-, tetra-, penta-, and hexanucleotides, respectively. Two SSRs separated by no more than 100 bp were treated as compound SSRs. Tandem repeats were analyzed using the tandem repeat finder (http://tandem.bu.edu/trf/trf.submit.options.html), with the following parameters: 2, 7, and 7 for match, mismatch and Indels, respectively; 50 and 500 for minimum alignment score to report repeat and maximum period size, respectively. Additionally, REPuter (http://bibiserv.techfak.uni-bielefeld.de/reputer/), with the minimal repeat size set to 30 bp and the Hamming distance set to 3, was used to identify dispersed repeats, including forward, palindromic, reverse, and complemented repeats.

### 2.3. Phylogenetic Analysis and Genome Comparison

All willow species with available cp genomes were included in the phylogenetic analysis.* Populus tremula* and* Populus trichocarpa* were used as the outgroup species. Phylogenetic trees based the whole cp genome sequences (genomic tree) and the coding sequences (CDS-tree) were constructed respectively. For the genomic tree, the complete cp genome sequences were first aligned using the MAFFT v7 [22], after which RAxML v8 was used to construct a maximum likelihood (ML) tree under the GTR+Γ model with 1000 bootstrap replicates [23]. The CDS-tree was generated using 55 protein-coding genes shared among the 18 species (16* Salix* species and 2* Populus* species). Specifically, ML trees for each gene were inferred separately with RAxML v8 [23], all of which were further used to infer the species tree with ASTRAL-III method [24]. The resulting species tree was visualized in FigTree 1.4.3 (http://tree.bio.ed.ac.uk/software/figtree/).

The mVISTA [25] was employed in the LAGAN mode to compare the cp genome of* S. wilsonii* with other* Salix* cp genomes. The annotation of* Salix arbutifolia* was used as a reference. The cp genomes for the following species were retrieved from the NCBI database:* S. arbutifolia* (NC_036718.1),* Salix babylonica* (NC_028350.1),* Salix chaenomeloides* (NC_037422.1),* Salix hypoleuca* (NC_037423.1),* Salix interior* (NC_024681.1),* Salix magnifica* (NC_037424.1),* Salix minjiangensis* (NC_037425.1),* Salix oreinoma* (NC_035743.1),* Salix paraplesia* (NC_037426.1),* Salix purpurea* (NC_029693.1),* Salix rehderiana* (NC_037427.1),* Salix rorida* (NC_037428.1),* Salix suchowensis* (NC_026462.1),* Salix taoensis* (NC_037429.1),* Salix tetrasperma* (NC_035744.1),* P. tremula* (NC_027425.1),* P. trichocarpa* (NC_009143.1),* Stockwellia quadrifida* (NC_022414.1), and* Oenothera elata* (NC_002693.2).

## 3. Results and Discussion

### 3.1. Assembly of the* S. wilsonii *cp Genome

A total of 42,633 chloroplast reads comprising 72,7581,388 nucleotides were extracted from the PacBio dataset. The reads were further filtered to remove repetitive and shorter sequences. Following correction and trimming, 505 PacBio RS reads were recovered containing 14,033,355 nucleotides (Table S1). The trimmed reads had a minimum length of 31,950 bp, a maximum length of 57,380 bp, and an N50 length of 35,004 bp. All of these reads were finally integrated into a complete circular pseudomolecule with a length of 155,750 bp long without any gap. The average depth of coverage of the* S. wilsonii* cp genome was approximately 90.1×.

**Figure 1:**
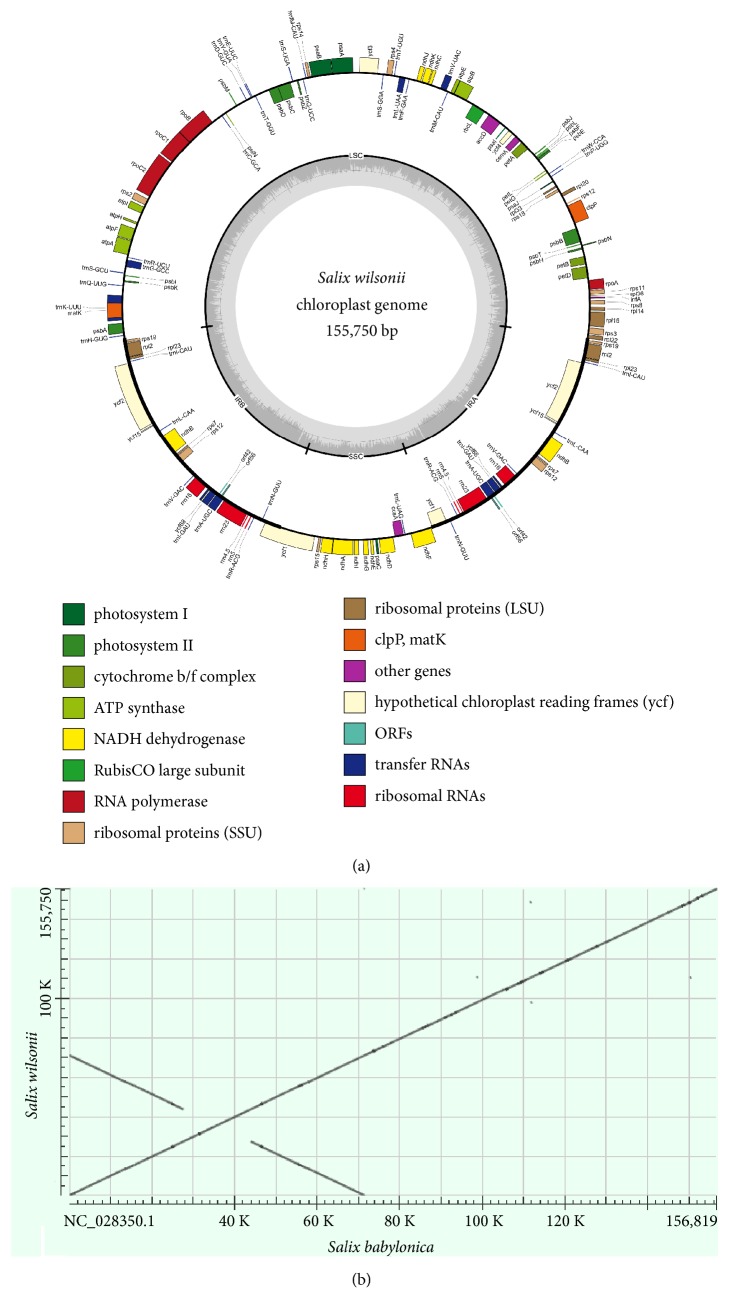
Assembly of* Salix wilsonii* cp genome. (a) Gene map of the chloroplast genome of* S. wilsonii*. (b) Dot matrix alignment of cp genomes between* S. wilsonii* and* S. babylonica*.

The size of the complete* S. wilsonii* cp genome was consistent with that of the cp genomes from the other sequenced* Salix* species (i.e., ranging from 154,977 bp in* S. magnifica* to 156,819 bp in* S. babylonica*) (Table S2). The assembled cp genome was a typical quadripartite molecule that included a pair of IRs (27,415 bp), an LSC region (84,638 bp), and an SSC region (16,282 bp) (Figure 1(a)). The overall GC content of the cp genome was 36.6%, and similar GC contents were calculated for the various willow species (Table S2). The GC content of the IR, LSC, and SSC region was 41.7%, 34.4%, and 31%, respectively. The observed higher GC content in the IR region was consistent with other angiosperm cp genomes [26, 27].

To evaluate the assembly quality,* S. wilsonii* and* S. babylonica* cp genome sequences were aligned according to an established dot matrix method [28]. The result revealed excellent collinearity between the two cp genomes, and neither inversion nor translocation was detected (Figure 1(b)), thus confirming the high quality of our assembly.

### 3.2. Cp Genome Annotation and Gene Loss Analysis

The chloroplasts of land plants generally contain approximately 100-120 unique genes [1]. In the* S. wilsonii* cp genome, 115 unique genes were predicted and divided into the following four categories: 78 protein-coding genes, 30 tRNA genes, four rRNA genes, and three pseudogenes (Table 1). The rRNA genes, seven tRNA genes (*trnA*-UGC,* trnI*-CAU,* trnI*-GAU,* trnL*-CAA,* trnN*-GUU,* trnR*-ACG, and* trnV*-GAC) and 10 protein-coding genes (*ndhB*,* rpl2*,* rpl23*,* rps7*,* rps19*,* ycf2*,* ycf15*,* pseudo-ycf68*,* orf42,* and* orf56*) were duplicated in the IR regions. The relatively high GC contents in the rRNA and tRNA genes explained why the highest GC content was detected in the IR region. Additionally, 58 protein-coding and 22 tRNA genes were located in the LSC region, whereas 10 protein-coding genes (*ccsA*,* ndhA*,* ndhD ndhE*,* ndhF*,* ndhG*,* ndhH*,* ndhI*,* psaC,* and* rps15*) and one tRNA gene (*trnL*-UAG) were present in the SSC region. The genes* rpl22* and* ycf1* spanned the boundary of IRb/LSC and IRa/SSC, respectively. A sequence analysis revealed that 50.05%, 1.81%, and 5.77% of the genome sequences encoded proteins, tRNAs and rRNAs, respectively. The remaining 42.37% comprised introns or intergenic spacers.

Two sets of ribosomal proteins, including 12 small ribosomal subunit proteins (encoded by* rps* genes) and nine large ribosomal subunit proteins (*rpl* genes), are commonly encoded in most plastid genomes [1]. We observed that two genes (*rps16* and* rpl32*) were missing from the* S. wilsonii* cp genome. Although plastomes rarely lose* rps* and* rpl* genes [1], the* rps16* and* rpl32* genes are missing throughout the family* Salicaceae*. Furthermore, BLAST homology searches of the* S. wilsonii* nuclear genome (unpublished data) with the corresponding gene sequences from the* Arabidopsis thaliana* cp genome (NC_000932.1) as queries (GeneIDs: 844798 for* rps16* and 844704 for* rpl32*) did not detect any fragments that matched these two genes. Thus, we suspected that* rps16* and* rpl32* were completely lost from the cell of* S. wilsonii*.

Two genes (*infA* and* ycf68*) were denoted as pseudogenes with truncated reading frames. The* infA *gene, which encodes the plastid translation initiation factor 1 (IF1), has been lost multiple times independently during the evolution of land plants and represents a classic example of chloroplast-to-nucleus gene transfer [29, 30]. The loss of* infA* was observed in the cp genomes of 11* Salicaceae* species as well [31]. A functional and intact* infA* is still retained in the spinach chloroplast with a length of 234 bp (encoding 77 residues) [29]. The* S. wilsonii pseudo-infA* (159 bp) was identified in the LSC region with part of the gene being absent (Figure S1(A)). A TBLASTN search using the intact spinach chloroplast IF1 as a query revealed a candidate gene encoding cp IF1 in the* S. wilsonii* nuclear genome (unpublished data). The identified nuclear gene was predicted to encode a protein of 146 residues, which contained a long N-terminal extension comparing with the IF1 encoded by spinach cp genome (Figure S1(B)). The elongated N-terminal is also observed in other angiosperms, and it has been demonstrated to function as a chloroplast-targeting signal in soybean and Arabidopsis [29]. The pseudogenization of cp-*infA* and the intact nuclear-encoded IF1 identified in* S. wilsonii* strongly suggested the transfer of the* infA* gene from the chloroplast to the nuclear genome, which is a general occurrence during angiosperm evolution [29, 30].

The hypothetical gene* ycf68*, located in the* trnI*-GAU intron, was first identified as ORF133b in* Oryza sativa* [32]. A comparative analysis indicated that the pine and grass lineage gained* ycf68* during the evolution of tracheophytes [33]. The* ycf68 *sequence is now considered as a cryptic reading frame that is widely conserved in several seed plants and liverwort species [34–37]. An alignment of the* ycf68* sequences among 14 angiosperms indicated that* ycf68* may be a functional protein-encoding gene in rice, corn, and* Pinus* species; however, in majority of cases, it is likely a nonfunctional gene because of numerous frameshifts and premature stop codons [34]. The cp genomes of* Salicaceae* species commonly carried sequences (approximately 380 bp) highly similar to the* ycf68* ORF in the* trnI*-GAU intron, but they were not previously annotated. The* S. wilsonii ycf68* sequence was highly homologous (92.45%) to the corresponding gene in* O. sativa *(NC_001320, Gene ID: 3131482), but many in-frame stop codons were found in the* pseudo-ycf68 *(Figure S2), resulting in a loss of function, which was consistent with the findings of previous studies [34, 36].

### 3.3. Codon Usage and Intron Loss Analysis

Based on the protein-coding genes, 25,899 codons were identified (excluding the stop codons). All genes had the canonical ATG start codon, except for* ndh*, which was started with ACG. The three most abundant amino acids were leucine (2,776; 10.72%), isoleucine (2,215; 8.55%), and serine (2,063; 7.97%), whereas cysteine (303; 1.17%) was the least abundant amino acid (Table 2). For amino acids coded by multiple codons, codon usage was biased toward A and U at the synonymous third position sites [38, 39], and a similar bias was observed in the* S. wilsonii* cp genome. Of the 29 preferred codons (RSCU > 1), 28 ended in an A or U. In contrast, among the 30 less frequently used codons (RSCU < 1), all but two ended in a G or C.

The tRNA and protein-coding genes of typical angiosperm cp genomes contain 17-20 Group II introns [40]. Of the 115 unique genes identified in the* S. wilsonii* cp genome, 14 contained one intron and three (*clpP*,* ycf3,* and* rps12*) contained two introns (Table 3), giving a total of 19 introns. The* rps12*, which encodes the 30S ribosomal protein S12, was a transspliced gene with the 5′-end located in the LSC region and the duplicated 3′-end located in the IR regions. The* trnK*-UUU had the largest intron (2,558 bp), which contained the* matK* gene, and the* petB* had the smallest intron (221 bp).

Although intron content is generally conserved among land plant cp genomes, there are several cases of intron gains or losses during evolution [2, 5, 40]. Guisinger et al. [39] described the loss of an intron from a tRNA gene (*trnG*-UCC) in photosynthetic angiosperms (*Geranium palmatum* and* Monsonia speciosa*). In the* S. wilsonii* cp genome, the* trnG*-UCC gene also lacked an intron. Moreover, by surveying all 15* Salix* cp genomes available in the NCBI database, we determined that the* trnG*-UCC intron, which appeared to be conserved across land plants [39], was absent from all willow cp genomes. The presence/absence of introns may provide valuable phylogenetic information and represents a potentially useful marker for resolving evolutionary relationships in many angiosperm lineages [41–43]. Therefore, future studies should clarify the distribution and phylogenetic utility of lost introns.

### 3.4. Repeat Sequence Analysis

Repeat sequences in cp genome contribute significantly to genomic structural variations, expansions, or rearrangements [1, 43]. An analysis of the repeat sequence in the* S. wilsonii* cp genome revealed 67 repeats, including 32 tandem repeats (sequence identity=100%) and 35 dispersed repeats (Table S3). The tandem repeat units were 11-25 bp long, and almost all of them were located in the intergenic spacer (IGS) regions. The three exceptions were located in the* rpl16* or* ycf3* intron regions. Among the dispersed repeats, 22 were forward repeats, two were reverse repeats, and 11 were palindromic repeats (Table S3). Most of the dispersed repeats were distributed in IGS regions, but some were detected in protein-coding genes.

Chloroplast simple sequence repeats (cpSSR) represent potentially useful markers for phylogenetic studies because of their haploid nature, relative lack of recombination, and uniparental inheritance [44]. We analyzed the type and distribution of SSRs in the* S. wilsonii* cp genome and detected 155 SSRs, including 118 perfect and 37 compound SSRs (Table 4). Among the perfect SSRs, there were 106, 1, 1, 8, and 2 for mono-, di-, tri-, tetra-, and pentanucleotide repeats, respectively. Hexanucleotide repeats were not detected. The longest repeat was 16 bp-stretch of A/T mononucleotides, and the major repeat unit was 8-10 bp (31 with 8 bp, 37 with 9 bp, and 18 with 10 bp), accounting for 72.9% (86/118) of all perfect SSRs. With one exception, all of the mononucleotide repeats consisted of A/T. Among the remaining 12 SSRs (repeat unit, 2-5 bp in length), seven contained only A and T bases (Table 4). The detection of AT-rich SSRs in the* S. wilsonii* cp genome was consistent with the findings in many other plant species [44]. The incidence of SSRs was proportional to the region size, with 110 in the LSC region, 18 in the IR region, and 27 in the SSC region. According to Ebert and Peakall [44], mononucleotide cpSSRs that located in a noncoding single copy (SC) region are more likely to exhibit intraspecies variation. We detected 94 mononucleotides distributed in noncoding SC regions of the* S. wilsonii* cp genome. These SSRs, together with the aforementioned tandem and dispersed repeats, may be useful for future ecological and evolutionary studies of willow species.

### 3.5. Inverted Repeat Contraction and Expansion

**Figure 2:**
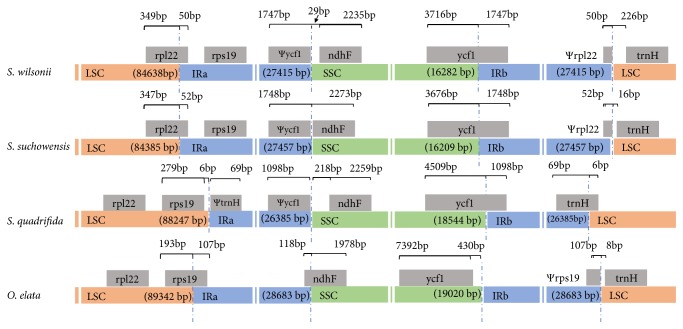
Comparison of IR boundaries among the cp genomes of four rosid plants. “Ψ” means pseudogene.

The IR regions, which are frequently subject to expansion, contraction, or even complete loss, play an important role for plastome stability and evolution [1, 45]. An examination of the IR boundary shifts may lead to a more thorough characterization of species-specific phylogenetic history. In this study, we compared the IR/SC boundaries of four rosid plants:* S. suchowensis*,* S. wilsonii*,* S. quadrifida*, and* O. elata*, which represent three different families (Figure 2).

The IR region length ranged from 26,385 bp to 28,683 bp, and some expansions/contractions of the IR regions were observed. Similar to most eudicot plastomes [46, 47], the IRa/LSC border in* O. elata* lied within the* rps19* gene, resulting in a* Ψrps19* (107 bp) at the IRb/LSC boundary (Figure 2). In* S. quadrifida*, the IRa region was detected adjacent to the* rps19* gene, and no pseudogene was detected at the IRb/LSC border. However, in both analyzed* Salix* species, the IRa/LSC junction expanded to partially include the* rpl22* gene, creating a* Ψrpl22* (approximately 50 bp) at the IRb/LSC boundary. The IRb/LSC junctions were located downstream of* trnH* (8-226 bp) in the examined species, except for the* S. quadrifida*, in which the* trnH* gene was incorporated in the IRb region, with 69 bp of this gene duplicated in the IRa region.

The IRb/SSC borders in both* Salix* species were located within* ycf1*. Thus, a* Ψycf1* was identified at the IRa/SSC border (1,747 bp in* S. wilsonii* and 1,748 bp in* S. suchowensis*). A portion of the* ndhF* gene reportedly overlapped with* Ψycf1* (140 bp) in* S. suchowensis* [48]. Moreover, the IRa/SSC border was located downstream of* ndhF* in* S. wilsonii* (29 bp). In* S. quadrifida*,* ycf1* also spanned the IRb/SSC junction; the IRa/SSC border was located downstream of* ndhF*, with 218 bp between* Ψycf1* and* ndhF*. Regarding* O. elata*, the IRa/SSC border was located within* ndhF* and the IRb/SSC boundary was located 430 bp from* ycf1*, which was inconsistent with the findings for most angiosperms [34, 47]. Changes in IR extent are the main factor affecting variations in overall plastome size and the number of genes [47]. Several elegant models have been proposed to explain the mechanisms underlying IR boundary shifts. These models involve gene conversions, double-strand breaks, and genomic deletions [49]. Future investigations should explore the conservation and evolutionary dynamics of the IR region among* Salicaceae* plants.

### 3.6. Phylogenetic Relationships and Comparative Analysis among* Salix* Species

**Figure 3:**
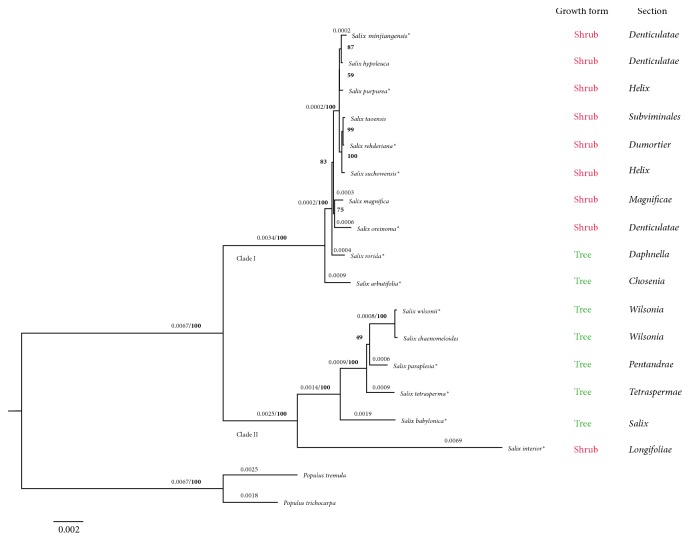
Maximum likelihood tree of willows and outgroups based on whole cp genome sequences. The branch length (≥0.0002) and the bootstrap value that supported each node (in bold) are shown above the branch. *∗* indicates the species selected for genome comparison analysis.

The taxonomy and phylogenetic relationships of the genus* Salix* based on morphology are extremely difficult due to the scarceness of informative morphological characters [50]. Furthermore, the dioecious reproduction and common interspecific hybridization of* Salix* species also complicate the traditional phenotypic characterization [50, 51]. The cp genomes have been proven highly effective for inferring the phylogenetic relationships in numerous plant groups. To elucidate the phylogenetic position of* S. wilsonii*, a ML tree was constructed based on the complete cp genome sequences of 16* Salix* species belonging to 13 different sections according to the Flora of China [16]. As shown in Figure 3, all the willow species were monophyletic and were evidently separated into two major clades with full support. The* S. wilsonii* and* S. chaenomeloides* from section* Wilsonia* formed a monophyletic group in Clade II. A CDS-tree based on was also constructed by using 55 protein-coding genes shared among the analyzed species The overall topology of the CDS-tree was very similar to that of the genomic tree; only some incongruence was found among the relationships of seven shrub willows, including* S. taoensis*,* S. hypoleuca*,* S. rehderiana*,* S. minjiangensis*,* S. purpurea*,* S. suchowensis,* and* S. magnifica* (Figure S3).

Although several molecular phylogenetic studies of the genus* Salix* have been published, most of them were carried out with nuclear internal transcribed spacers or a few chloroplast DNA regions [15, 50, 52–54]. Two phylogenetic analyses focused on the relationships of the genera* Salix* and* Populus* were recently conducted with the chloroplast protein-coding gene dataset and complete cp genome, respectively [31, 55]. Considering the limited number of* Salix* species involved in Huang et al.'s study [31], we compared the relationships resolved in the genomic tree with those reported by Zhang et al. [55]. Overall, the two main clades formed within the genus* Salix* were generally consistent, but some inconsistences were observed among the interspecific relationships in each clade. These inconsistencies may have been due to the use of different datasets during the phylogenetic analysis, since the phylogenetic relationships presented in Clade I of our CDS-tree (Figure S3) were almost the same with that of Zhang et al.

**Figure 4:**
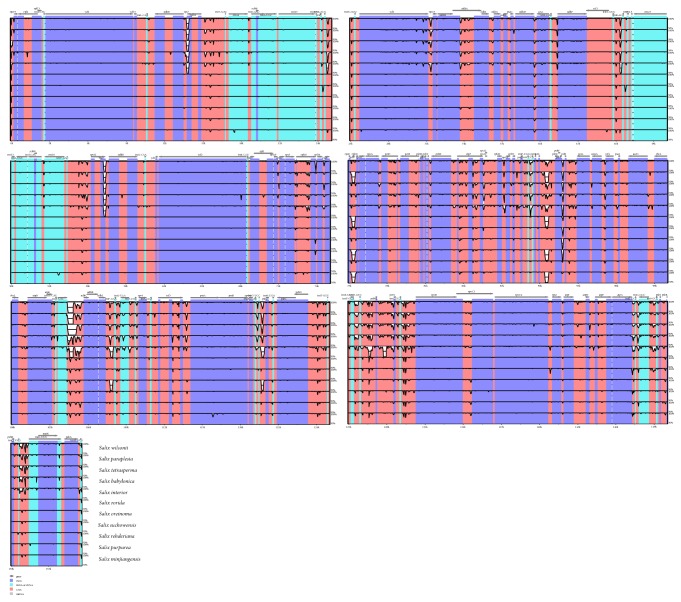
Complete chloroplast genome comparison of 12* Salix* species using mVISTA program with* S. arbutifolia* as a reference. Cp genome regions are color-coded as protein-coding (exon), rRNA, tRNA, and conserved noncoding sequences (CNS).

In order to compare the sequence variation within the genus* Salix*, the whole cp genomes of 12* Salix* species were aligned using mVISTA with* S. arbutifolia* as a reference (Figure 4). The results revealed high sequence similarity across the willow cp genomes. Consistent with other angiosperms [56, 57], the IR regions were more conserved than the LSC and SSC regions, and the noncoding regions were more divergent than the coding regions. Based on the alignment, the highly divergent regions were detected in the IGS regions:* ycf1*-*rps15*, *trnN*^GUU^-*trnR*^*ACG*^, *trnV*^GAC^-*rps12*,* rps7*-*ndhB*,* rpl14-rps8*,* rps8-infA*,* rpoA-petD*,* psbB-clpP*,* rpl20-rpl18*,* rpl33-psaJ*,* trnP*^*UGG*^*-trnW*^*CCA*^,* petL-psbE*,* psbL-petA*,* cemA-ycf4*,* ycf4*-*psaI*,* rbcL*-*accD*,* trnV*^*UAC*^*-ndhC*,* ndhJ-trnF*^*GAA*^,* trnL*^*UAA*^*-trnT*^*UGU*^,* trnT*^*UGU*^*-rps4*,* ycf3-psaA*,* trnfM-trnG*^*UCC*^,* trnG*^*UCC*^*-psbZ*,* psbD-TrnT*^*GGU*^,* trnY*^*GUA*^*-trnD*^*GUC*^,* trnD*^*GUC*^*-psbM*,* psbM-psbN*,* trnC*^*GCA*^*-rpoB*,* trnG*^*GCC*^-*trnSGCU*,* trnQ*^*UUG*^-*trnK*^*UUU*^, and* psbA*-*trnH*^*GUG*^. For the coding regions, the more divergent regions were found in* rps7*,* ycf1,* and* matK*. These highly variable regions can be used to develop more informative DNA barcodes and facilitate phylogenic analysis among* Salix* species.

## 4. Conclusions

In this study, we assembled and characterized the complete cp genome of* S. wilsonii*, which is an endemic and ornamental willow tree in China. The* S. wilsonii* cp genome was structurally and organizationally similar to the cp genomes of other* Salix* species. Significant shifts in the IR boundaries were revealed in comparison with the cp genomes from three other rosid plant species. An analysis of the phylogenetic relationships among 16 willow species indicated* S. wilsonii* and* S. chaenomeloides* were sister species and revealed the monophyly of the genus* Salix*. The complete* S. wilsonii* cp genome represents a useful sequence-based resource which can be further applied for phylogenetic and evolutionary studies in woody plants.

## Figures and Tables

**Table 1:** Genes present in the cp genome of *Salix wilsonii*.

Gene category	Group of genes	Name of genes				
Self-replication	Ribosomal RNA genes	*rrn16*	*rrn23*	*rrn4.5*	*rrn5*	
	Transfer RNA genes	*trnA-*UGC	*trnC-*GCA	*trnD-*GUC	*trnE-*UUC	*trnF-*GAA
		*trnfM-*CAU	*trnG-*GCC	*trnG-*UCC	*trnH*-GUG	*trnI-*CAU
		*trnI-*GAU	*trnK-*UUU	*trnL-*CAA	*trnL-*UAA	*trnL-*UAG
		*trnM-*CAU	*trnN-*GUU	*trnP-*UGG	*trnQ-*UUG	*trnR-*ACG
		*trnR-*UCU	*trnS-*GCU	*trnS-*GGA	*trnS-*UGA	*trnT-*GGU
		*trnT-*UGU	*trnV-*GAC	*trnV-*UAC	*trnW-*CCA	*trnY-*GUA
	Large subunit of ribosome (LSU)	*rpl2*	*rpl14*	*rpl16*	*rpl20*	*rpl22*
		*rpl23*	*rpl33*	*rpl36*		
	Small subunit of ribosome (SSU)	*rps2*	*rps3*	*rps4*	*rps7*	*rps8*
		*rps11*	*rps12*	*rps14*	*rps15*	*rps18*
		*rps19*				
	RNA polymerase	*rpoA*	*rpoB*	*rpoC1*	*rpoC2*	

Genes for photosynthesis	Photosystem h	*psaA*	*psaB*	*psaC*	*psaI*	*psaJ*
	Photosystem II	*psbA*	*psbB*	*psbC*	*psbD*	*psbE*
		*psbF*	*psbH*	*psbI*	*psbJ*	*psbK*
		*psbL*	*psbM*	*psbN*	*psbT*	*psbZ*
	Cytochrome b/f complex	*petA*	*petB*	*petD*	*petG*	*petL*
		*petN*				
	ATP synthase	*atpA*	*atpB*	*atpE*	*atpF*	*atpH*
		*atpI*				
	ATP-dependent protease subunit p	*clpP*				
	Large subunit of rubisco	*rbcL*				
	NADH dehydrogenase	*ndhA*	*ndhB*	*ndhC*	*ndhD*	*ndhE*
		*ndhF*	*ndhG*	*ndhH*	*ndhI*	*ndhJ*
		*ndhK*				

Other genes	Maturase	*matK*				
	Envelop membrane protein	*cemA*				
	Subunit of acetyl-CoA-carboxylase	*accD*				
	C-type cytochrome synthesis gene	*ccsA*				

Unknown function	Hypothetical chloroplast reading frames	*ycf1*	*ycf2*	*ycf3*	*ycf4*	*ycf15*
		*orf42*	*orf56*			
	Pseudogene	*pseudo-infA*	*pseudo-ycf68*	*pseudo-ycf1*		

**Table 2:** The relative synonymous codon usage in the *Salix wilsonii* cp genome.

Amino	Codon	Number	RSCU^*∗*^	Amino	Codon	Number	RSCU^*∗*^
acid				acid			
Ala	GCU	614	1.83	Leu	UUA	843	1.82
	GCA	371	1.11		CUU	578	1.25
	GCC	210	0.63		UUG	568	1.23
	GCG	146	0.44		CUA	393	0.85
Asn	AAU	949	1.52		CUC	207	0.45
	AAC	299	0.48		CUG	187	0.4
Asp	GAU	808	1.57	Lys	AAA	972	1.44
	GAC	223	0.43		AAG	374	0.56
Arg	AGA	482	1.87	Met	AUG	622	1
	CGA	355	1.38	Phe	UUU	979	1.29
	CGU	321	1.24		UUC	543	0.71
	AGG	164	0.64	Pro	CCU	424	1.56
	CGG	117	0.45		CCA	307	1.13
	CGC	109	0.42		CCC	202	0.74
Cys	UGU	208	1.37		CCG	157	0.58
	UGC	95	0.63	Ser	UCU	574	1.67
Gln	CAA	669	1.49		AGU	408	1.19
	CAG	228	0.51		UCA	404	1.17
Gly	GGA	706	1.58		UCC	341	0.99
	GGU	554	1.24		UCG	200	0.58
	GGG	333	0.75		AGC	136	0.4
	GGC	194	0.43	Thr	ACU	528	1.59
Glu	GAA	1003	1.48		ACA	413	1.25
	GAG	352	0.52		ACC	248	0.75
His	CAU	471	1.51		ACG	136	0.41
	CAC	151	0.49	Trp	UGG	449	1
Ile	AUU	1091	1.48	Tyr	UAU	782	1.64
	AUA	682	0.92		UAC	174	0.36
	AUC	442	0.6	Val	GUA	532	1.52
					GUU	500	1.43
					GUG	202	0.58
					GUC	169	0.48

Note: *∗* relative synonymous codon usage, RSCU.

**Table 3:** Genes with introns in the cp genome of *Salix wilsonii*.

Gene	Location	Exon I	Intron I	Exon II	Intron II	Exon III
		(bp)	(bp)	(bp)	(bp)	(bp)
*atpF*	LSC	145	731	410		
*clpP*	LSC	69	829	291	598	228
*ndhA*	SSC	564	1074	546		
*ndhB*	IR	777	682	756		
*petB*	LSC	5	221	643		
*petD*	LSC	9	782	489		
*rpl2*	IR	399	629	471		
*rpl16*	LSC	9	1114	402		
*rpoC1*	LSC	453	779	1617		
*rps12*	trans	114	-	231	537	30
*trnA*-UGC	IR	38	800	35		
*trnG-GCC*	LSC	23	703	48		
*trnI*-GAU	IR	37	947	35		
*trnK*-UUU	LSC	37	2558	29		
*trnL*-UAA	LSC	37	583	50		
*trnV*-UAC	LSC	39	607	37		
*ycf3*	LSC	129	722	228	716	153

**Table 4:** Numbers of SSRs identified in the cp genome of *Salix wilsonii*.

SSR repeat type	SSR repeat unit	Number of repeats														Total
		3	4	5	6	7	8	9	10	11	12	13	14	15	16	
Monomer	A/T						31	36	18	9	4	3	2	1	1	105
	C/G							1								1
Dimer	TA									1						1
Tripolymer	TAT		1													1
Tetramer	AATG	1														1
	AGAA	1														1
	TAGA	1														1
	TATT	1														1
	TTCA	1														1
	TTTA	2														2
	TTTC	1														1
Pentamer	AATTT	1														1
	ATTAA	1														1
Compound																37
Total																155

## Data Availability

The cp genome data used to support the study findings are included in the article.

## Supplementary Materials

Supplementary 1 Table S1: statistics for the assembly of the* Salix wilsonii* cp genome.

Supplementary 2 Table S2: features of chloroplast genomes from 16* Salix* species.

Supplementary 3 Table S3: statistics of repeat sequences in the* Salix wilsonii* cp genome.

Supplementary 4 Figure S1: pairwise and multiple sequence alignment. (A) Pairwise alignment of the* infA* genes from cp genomes of* Spinacia oleracea* (AF206521) and* Salix wilsonii*. (B) Multiple alignment of IF1 protein sequences. The accession numbers for these proteins are NP_192856.1 (*Arabidopsis thaliana*), AAK38870.1 (soybean,* Glycine max*), NP_054969 (spinach,* Spinacia oleracea*), and* Salix wilsonii* (EVM0016759.1, unpublished data). cp, chloroplast; nuc, nuclear.

Supplementary 5 Figure S2: alignment of the* ycf68* genes from* Oryza sativa* and* Salix wilsonii*. Red boxes indicate in-frame stop codons.

Supplementary 6 Figure S3: the phylogenetic tree based on 55 protein-coding genes of 16* Salix* and two* Populus* species. The branch length was shown on the branch and the branch support value (in bold) was shown at the node.
