# Supplementary material for: Analyzing and Characterizing the Chloroplast Genome of Salix wilsonii
Source: Biomed Res Int. 2019 Jul 15;2019:5190425. doi: 10.1155/2019/5190425 (PMC6662467; doi:10.1155/2019/5190425)
Supplement: Supplementary 1 — Table S1: statistics for the assembly of the Salix wilsonii cp genome. [file 5190425.f1.docx]

Table S1 Statistics for the assembly of the Salix wilsonii cp genome

| Process | Reads no. | Total length  (bp) |
| --- | --- | --- |
| Input | 42,633 | 727,581,388 |
| Filtered Output | 9,704 | 203,283,367 |
| Correction Output | 505 | 14,092,368 |
| Trimming Output | 505 | 14,033,355 |
