# Supplementary material for: Analyzing and Characterizing the Chloroplast Genome of Salix wilsonii
Source: Biomed Res Int. 2019 Jul 15;2019:5190425. doi: 10.1155/2019/5190425 (PMC6662467; doi:10.1155/2019/5190425)
Supplement: Supplementary 2 — Table S2: features of chloroplast genomes from 16 Salix species. [file 5190425.f2.docx]

Table S2 Comparison of chloroplast genomes among 16 species of *Salix*

| Growth  form | Species  (Accsession no.) | Genome size (bp) | IR (bp) | LSC (bp) | SSC (bp) | GC content (%) | No. of unique gene | No. of unique protein gene | No. of unique tRNA gene | No. of unique rRNA gene | No. of pseudogene |
| --- | --- | --- | --- | --- | --- | --- | --- | --- | --- | --- | --- |
| Shrub  willow | *Salix hypoleuca*  (NC_037423) | 155126 | 27458 | 16235 | 83975 | 36.7 | 107 | 74 | 29 | 4 | NA |
|  | *Salix interior*  (NC_024681) | 156620 | 27167 | 16306 | 85980 | 37.0 | 113 | 77 | 30 | 4 | 2 |
|  | *Salix magnifica*  (NC_037424) | 154977 | 27458 | 16204 | 83857 | 36.7 | 107 | 74 | 29 | 4 | NA |
|  | *Salix minjiangensis*  (NC_037425) | 155038 | 27449 | 16215 | 83925 | 36.7 | 108 | 74 | 30 | 4 | NA |
|  | *Salix oreinoma*  (NC_035743) | 155531 | 27427 | 16211 | 84466 | 36.7 | 113 | 77 | 30 | 4 | 2 |
|  | *Salix purpurea*  (KP019639) | 155590 | 27459 | 16220 | 84452 | 36.7 | 111 | 76 | 30 | 4 | 1 |
|  | *Salix rehderiana*  (NC_037427) | 155051 | 27459 | 16219 | 83914 | 36.7 | 107 | 74 | 29 | 4 | NA |
|  | *Salix suchowensis*  (NC_026462) | 155214 | 27459 | 16219 | 84077 | 36.7 | 111 | 76 | 30 | 4 | 1 |
|  | *Salix taoensis*  (NC_037429) | 155085 | 27459 | 16220 | 83947 | 36.7 | 107 | 74 | 29 | 4 | NA |
| Tree willow | *Salix arbutifolia*  (NC_036718) | 155661 | 27455 | 16215 | 84536 | 36.7 | 115 | 79 | 30 | 4 | 2 |
|  | *Salix babylonica*  (NC_028350) | 156819 | 27667 | 16231 | 85254 | 36.6 | 112 | 77 | 30 | 4 | 1 |
|  | *Salix chaenomeloides*  (NC_037422) | 156154 | 27287 | 16302 | 85278 | 36.7 | 108 | 74 | 29 | 4 | 1 |
|  | *Salix paraplesia*  (NC_037426) | 155553 | 27455 | 16303 | 84340 | 36.7 | 108 | 74 | 29 | 4 | 1 |
|  | *Salix rorida*  (NC_037428) | 155144 | 27460 | 16213 | 84011 | 36.7 | 107 | 74 | 29 | 4 | NA |
|  | *Salix tetrasperma*  (NC_035744) | 155671 | 27399 | 16289 | 84584 | 36.7 | 113 | 77 | 30 | 4 | 2 |
|  | *Salix wilsonii* | 155750 | 27415 | 84638 | 16282 | 36.6 | 115 | 78 | 30 | 4 | 3 |

Note: The shaded numbers were referred to Huang et al., (2017). NA: information not available.
