# Supplementary material for: Analyzing and Characterizing the Chloroplast Genome of Salix wilsonii
Source: Biomed Res Int. 2019 Jul 15;2019:5190425. doi: 10.1155/2019/5190425 (PMC6662467; doi:10.1155/2019/5190425)
Supplement: Supplementary 5 — Figure S2: alignment of the ycf68 genes from Oryza sativa and Salix wilsonii. Red boxes indicate in-frame stop codons. [file 5190425.f5.docx]

Oryza 1 ATGGCGTACTCCTCCTGTTTGAATCGGAGTTTGAAACCAAACAAACTTCT 50

||||||||||.||||||||.|||.|||.||||||||| |||||||||

Salix 1 ATGGCGTACTTCTCCTGTTCGAACCGGGGTTTGAAAC----CAAACTTCT 46

Oryza 51 CCTCAGGAGGATAGATGGGGCGATTCAGGTGAGATCCCATGTAGATCTAA 100

|||||||||||||||||||||||||||||||||||||.|||||||||.||

Salix 47 CCTCAGGAGGATAGATGGGGCGATTCAGGTGAGATCCAATGTAGATCCAA 96

*Oryza* 101 CTTTCTATTCACTCGTGGGATCCGGGCGGTCCGGGGGGGGCACTACGGCT 150

||||||||||||||||||||||||||||||||||||||..|||.|.||||

*Salix* 97 CTTTCTATTCACTCGTGGGATCCGGGCGGTCCGGGGGGACCACCATGGCT 146

*Oryza* 151 CCTCTCTTCTCGAGAATCCATACATCCCTTATCAGTGTATGGAGAGCTAT 200

|||||||||||||||||||||||||||||||||||||||||||.||||||

*Salix* 147 CCTCTCTTCTCGAGAATCCATACATCCCTTATCAGTGTATGGACAGCTAT 196

*Oryza* 201 CTCTCGAGCACAGGTTGAGGTTCGTCCTCAATGGG-----AAAATGGAGC 245

||||||||||||||||.|||||||.|||||||||| ||||||||||

*Salix* 197 CTCTCGAGCACAGGTTTAGGTTCGGCCTCAATGGGAAAATAAAATGGAGC 246

*Oryza* 246 ACCTAACAACGCATCTTCACAGACCAAGAACTACGAGATC-ACCCTTTCA 294

|||||||||||.|||||||||||||||||||||||||||| .||||||||

*Salix* 247 ACCTAACAACGTATCTTCACAGACCAAGAACTACGAGATCGCCCCTTTCA 296

*Oryza* 295 TTCTGGGGTGACGGAGGGATCGTACCATTCGAGCC-TTTTTTTCATGCTT 343

||||||||.|||||||||||||||||||||||||| ||||||||||||||

*Salix* 297 TTCTGGGGCGACGGAGGGATCGTACCATTCGAGCCTTTTTTTTCATGCTT 346

*Oryza* 344 TTCCCGGCGGTCTGGAGAAAGCAGCAATCAATAGGACTTCCCTAATCCTC 393

|||||||.||||||||||||||.|||||||||||

*Salix* 347 TTCCCGGAGGTCTGGAGAAAGCTGCAATCAATAG---------------- 380

*Oryza* 394 CCTTCCTGA 402

*Salix* 381 --------- 380

Figure S2 Alignment of the *ycf68* gene between *Oryza sativa* and *Salix wilsonii*. Codons highlighted in red boxes represent in frame stop codons.
