# Supplementary figures and images for: Analyzing and Characterizing the Chloroplast Genome of Salix wilsonii
Source: Biomed Res Int. 2019 Jul 15;2019:5190425. doi: 10.1155/2019/5190425 (PMC6662467; doi:10.1155/2019/5190425)

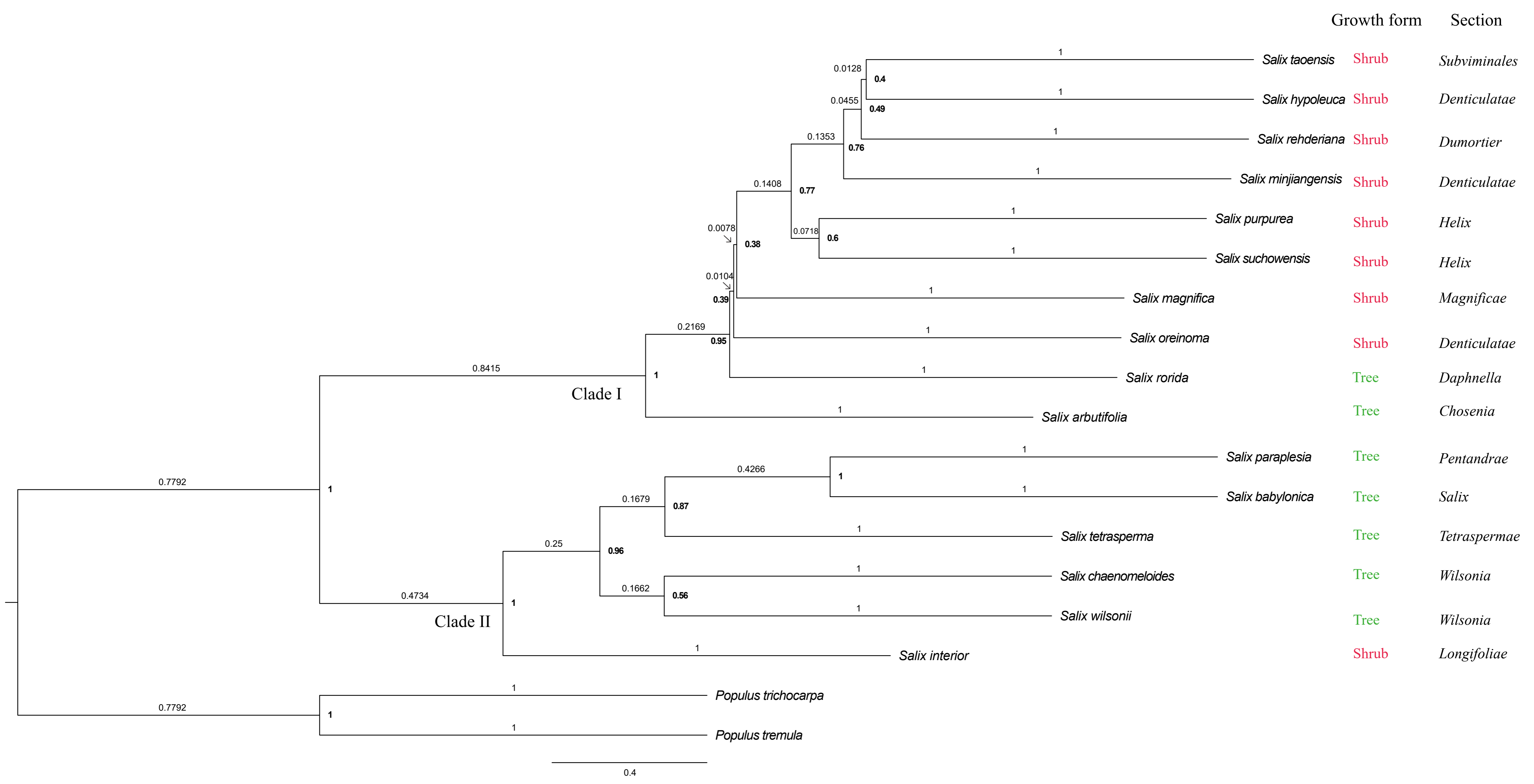

Supplement: Supplementary 6 — Figure S3: the phylogenetic tree based on 55 protein-coding genes of 16 Salix and two Populus species. The branch length was shown on the branch and the branch support value (in bold) was shown at the node. [file 5190425.f6.pdf]
